# Supplementary material for: Fish primary embryonic pluripotent cells assemble into retinal tissue mirroring in vivo early eye development
Source: eLife. 2021 Jul 12;10:e66998. doi: 10.7554/eLife.66998 (PMC8275126; doi:10.7554/eLife.66998)
Supplement: Transparent reporting form [file elife-66998-transrepform.docx]

***eLife’s* transparent reporting form**

We encourage authors to provide detailed information *within their submission* to facilitate the interpretation and replication of experiments. Authors can upload supporting documentation to indicate the use of appropriate reporting guidelines for health-related research (see [EQUATOR Network](http://www.equator-network.org/%20)), life science research (see the [BioSharing Information Resource](https://biosharing.org/)), or the [ARRIVE guidelines](http://www.plosbiology.org/article/info:doi/10.1371/journal.pbio.1000412) for reporting work involving animal research. Where applicable, authors should refer to any relevant reporting standards documents in this form.

If you have any questions, please consult our Journal Policies and/or contact us: [editorial@elifesciences.org](mailto:editorial@elifesciences.org).

**Sample-size estimation**

- You should state whether an appropriate sample size was computed when the study was being designed
- You should state the statistical method of sample size computation and any required assumptions
- If no explicit power analysis was used, you should describe how you decided what sample (replicate) size (number) to use

Please outline where this information can be found within the submission (e.g., sections or figure legends), or explain why this information doesn’t apply to your submission:

Replicates numbers were decided from experience and practical considerations on the techniques performed. No prior power analysis was used as the results were consistent between different samples, experiments and individuals performing experiments. N numbers are given in every figure legend, statistical tests are described in the Materials and Methods.

**Replicates**

- You should report how often each experiment was performed
- You should include a definition of biological versus technical replication
- The data obtained should be provided and sufficient information should be provided to indicate the number of independent biological and/or technical replicates
- If you encountered any outliers, you should describe how these were handled
- Criteria for exclusion/inclusion of data should be clearly stated
- High-throughput sequence data should be uploaded before submission, with a private link for reviewers provided (these are available from both GEO and ArrayExpress)

Please outline where this information can be found within the submission (e.g., sections or figure legends), or explain why this information doesn’t apply to your submission:

The number of biological and technical (different day of experiment) replicas are given in figure legend.

1) We generated about 1500 organoids from medaka embryos from 52 independent experiments and about 300 organoids from zebrafish embryos from about 5 independent experiments.

The efficiency of medaka cell aggregation was followed in 84 aggregates in two biological experiments.

2) Onset of Rx3 expression was followed in 71 organoids in two biological experiments. Analysis of the onset of Rx3 expression was performed from one technical experiment each - medaka and zebrafish. Medaka: n=6 embryos, n=17 organoids. Zebrafish=6 embryos, n=15 organoids.

3) Phenotypic analysis in medaka Rx3KO was performed in one technical experiment with n=13 organoids.

4) Phenotypic analysis in regular (>1,000 cells) versus small (<1,000 cells) organoids was performed and analyzed in 10 technical experiments on 2 genetic backgrounds (wild-type and *Rx3::H2B-GFP*) with about 10 organoids each.

5) Cell tracking was performed on 3 biological experiments. Type and directionality of migration was analyzed on 1 dataset

We have not encountered outliers. No data was excluded from the analysis.

**Statistical reporting**

- Statistical analysis methods should be described and justified
- Raw data should be presented in figures whenever informative to do so (typically when N per group is less than 10)
- For each experiment, you should identify the statistical tests used, exact values of N, definitions of center, methods of multiple test correction, and dispersion and precision measures (e.g., mean, median, SD, SEM, confidence intervals; and, for the major substantive results, a measure of effect size (e.g., Pearson's r, Cohen's d)
- Report exact p-values wherever possible alongside the summary statistics and 95% confidence intervals. These should be reported for all key questions and not only when the p-value is less than 0.05.

Please outline where this information can be found within the submission (e.g., sections or figure legends), or explain why this information doesn’t apply to your submission:

In every experiment, we compared differences between two independent groups (embryo versus organoid, small (<1,000 cells) versus regular (>1,000 cells) size), we used Wilcoxon-Mann-Whitney two-sample rank test.

Maximum projections from live imaging of organoids (n=3) presented in Figure 5, Video 5 and Video 6. Corresponding Raw data is available in public repository as stated in Materials and Methods.

All numerical data is presented as mean and standard deviation.

All statistical data reported as ns for 0.05<p<1, * for 0.01<p<0.05, ** for 0.001<p<0.01, *** 0.0001<p<0.001, **** for p<0.0001

(For large datasets, or papers with a very large number of statistical tests, you may upload a single table file with tests, Ns, etc., with reference to sections in the manuscript.)

**Group allocation**

- Indicate how samples were allocated into experimental groups (in the case of clinical studies, please specify allocation to treatment method); if randomization was used, please also state if restricted randomization was applied
- Indicate if masking was used during group allocation, data collection and/or data analysis

Please outline where this information can be found within the submission (e.g., sections or figure legends), or explain why this information doesn’t apply to your submission:

Samples were allocated into experimental or control groups depending on their origin (embryo/organoid), size (>1,000 cells or <1,000 cells) and genetic background (Rx3, Atoh7).

**Additional data files (“source data”)**

- We encourage you to upload relevant additional data files, such as numerical data that are represented as a graph in a figure, or as a summary table
- Where provided, these should be in the most useful format, and they can be uploaded as “Source data” files linked to a main figure or table
- Include model definition files including the full list of parameters used
- Include code used for data analysis (e.g., R, MatLab)
- Avoid stating that data files are “available upon request”

Please indicate the figures or tables for which source data files have been provided:

Raw datasets from time-lapse experiments (Figure 5, Video 5) are deposited in publically available repository; <https://heidata.uni-heidelberg.de/> (https://heidata.uni-heidelberg.de/dataset.xhtml?persistentId=doi%3A10.11588%2Fdata%2FAOSUS8&version=DRAFT).

Matlab script for analysis for directionality is available on GitHub (https://github.com/VeneraW/DirectionalityAnalysisOrganoids) as stated in Materials and Methods.
